# Transcriptomic Analysis of Yunwu Tribute Tea Leaves under Cold Stress

**DOI:** 10.3390/cimb45010047

**Published:** 2023-01-13

**Authors:** Ying Wang, Cheng Wan, Leijia Li, Zhun Xiang, Jihong Wang, Yan Li, Degang Zhao

**Affiliations:** 1 Guizhou Province Institute of Biology, Guizhou Academy of Sciences, Guiyang 550009, China; 2 The Key Laboratory of Plant Resources Conservation and Germplasm Innovation in Mountainous Region (Ministry of Education), Guizhou University, Guiyang 550025, China; 3 The Application Center for Plant Conservation Technology, Guizhou Academy of Agricultural Sciences, Guiyang 550006, China

**Keywords:** transcriptome, cold stress, metabolic pathways, *Camellia sinensis* (L.) Kuntze var. *niaowangensis* Q. H. Chen

## Abstract

Background: Cold stress usually occurs in winter and is one of the most significant environmental factors restricting the growth of the tea plant as well as its geographical distribution. Objective: It is necessary to identify the physiological and molecular mechanisms of plants under cold stress so that cold-tolerant crop varieties can be cultivated to limit production losses. At the same time, this would allow the crop planting area to be expanded, hence improving the economic benefits. Methods: In this study, the transcriptome data of Yunwu Tribute Tea under cold conditions were obtained using the Illumina HiSeq platform. By analyzing changes in transcriptome data associated with the antioxidant enzyme system, plant hormone signal transduction, proline and tyrosine metabolism pathways, and transcription factors, the molecular mechanisms involved in Yunwu Tribute Tea under cold stress were investigated. Results: In this study, Illumina HiSeq technology was applied to investigate the cold-tolerance mechanism. For this purpose, cDNA libraries were obtained from two groups of samples, namely the cold-treated group (DW) and the control group (CK). A total of 185,973 unigenes were produced from 511,987 assembled transcripts; among these, 16,020 differentially expressed genes (DEGs) (corrected *p*-value < 0.01, |log2(fold change)| >3), including 9606 up-regulated and 6414 down-regulated genes, were obtained. Moreover, the antioxidant enzyme system, plant hormone signal transduction, proline and tyrosine metabolism pathways, and transcription factors were analyzed; based on these results, a series of candidate genes related to cold stress were screened out and discussed. The physiological indexes related to the low-temperature response were tested, along with five DEGs which were validated by quantitative real-time PCR. Conclusions: Differential gene expression analysis has confirmed that substantial cold-responsive genes are related to the antioxidant enzyme system, plant hormone signal transduction, proline metabolism pathway, tyrosine metabolism pathway, and transcription factors.

## 1. Introduction

Yunwu Tribute Tea [*Camellia sinensis* (L.) Kuntze var. *niaowangensis* Q. H. Chen] has a long history of cultivation, dating back to as early as the Tang Dynasty. This was recorded by Lu Yu, the Saint of Tea, and it began to be used within the imperial court in 1325, before becoming a royal treasure in the Ming and Qing Dynasties. In addition, it was listed as one of the eight most famous teas in China during the Qianlong period. More recently, Guiding Snow Bud produced from Yunwu Tribute Tea won international gold awards to become an internationally famous tea. In fact, it is the only famous tea recorded in the tribute tea monument of China [1]. 

When plants are subjected to low-temperature stress, their cell membrane system is destroyed, the absorption of mineral nutrients is affected, biosynthesis is reduced, the absorption of light energy is reduced, and many other physiological functions are blocked, resulting in permanent tissue damage and even plant death [2,3]. Most plants, especially some important cash crops, are sensitive to low temperature as once freezing occurs, their survival rate decreases, overwintering becomes difficult, growth is restricted, and output is greatly reduced, thereby impacting agricultural and forestry production as well as the associated economic benefits. In southwest China, a typical mountainous and hilly agricultural area at high altitude, differences between winter and summer are great. Frost and snow are significant in winter, and since most crops either grow slowly or show high mortality during this period, the development of local agriculture and animal husbandry is severely affected. Through long-term evolution processes, plants have gradually developed a complex and efficient response mechanism to the cold, especially in terms of changes in their phenotypic structures, physical and chemical properties, and cellular and molecular functions which allow them to cope with low-temperature stress. Cold tolerance can also be improved through domestication; however, none of these two methods can solve the problem of cold injury in plants. Therefore, it is necessary to identify the physiological and molecular mechanisms of plants under cold stress so that cold-tolerant crop varieties can be cultivated to limit production losses. At the same time, this would allow the crop planting area to be expanded, hence improving the economic benefits.

In recent years, researchers have made great progress in the study of plant cold tolerance through RNA-seq, Chip-seq, iTRAQ, TMT, LC-MS/MS and other new generation technologies that have developed. As a result, it has become possible to analyze the mechanism of cold tolerance in plants at the transcriptome, proteome, metabolome and transcriptional–translational regulation levels. This, in turn, has helped to identify a large number of cold tolerance genes, the knowledge of which could be applied to improve cold tolerance of plants through molecular and transgenic breeding techniques, or even to improve the breeding efficiency of cold-tolerant varieties. High-throughput sequencing technologies, such as Illumina-based second-generation sequencing platforms or third-generation sequencing PacBio, have led to unprecedented advances in genome research.

In this study, the transcriptome data of Yunwu Tribute Tea under cold conditions were obtained using the Illumina HiSeq platform (Illumina, San Diego, CA, USA). By analyzing changes in transcriptome data associated with the antioxidant enzyme system, plant hormone signal transduction, proline metabolism pathway, tyrosine metabolism pathway, and transcription factors, the molecular mechanism involved in Yunwu Tribute Tea under cold stress was clarified.

## 2. Materials and Methods

### 2.1. Stress Treatment of Experimental Materials

The test materials consisted of 1-year-old seedlings of Yunwu Tribute Tea, taken from the nursery of the Tea Industry Office of the Agricultural Bureau of Yunwu Town, Guiding County, Guizhou Province, China. Based on the analysis of their physiological and biochemical indexes under low-temperature stress [4]. the critical temperature and stress time of the Yunwu Tribute Tea leaves were found to be 1 °C for 3 days. For the cold-treated group (DW), the light intensity was kept at 10,000 lux, the relative humidity at 50%, and the photoperiod at 16 h/8 h of day/night. Similar conditions were used for the control group (CK), except that the temperature was kept at 25 °C.

The upper leaves were harvested from different seedlings (for both the DW and the CK groups) for material homogeneity and the samples were immediately frozen in liquid nitrogen for storage at −80 °C. Three biological replicates per treatment were then used for the subsequent RNA extraction and analysis.

### 2.2. RNA Extraction and Qualification

Total RNA was extracted from the samples by first adding 1000 mL of TRIzol (Invitrogen, Carlsbad, CA, USA) before mixing and incubation at room temperature for 30 min. A volume of 200 mL of chloroform was then added and, once the samples were vortexed and incubated at room temperature for 10 min, they were centrifuged at 12,000× *g* for 15 min at 2–8 °C. After centrifugation, 400 mL of the supernatant was transferred to a clean 1.5 mL tube, to which 500 mL of isopropanol was added. The tube was inverted several times and after overnight incubation at 4 °C, the samples were centrifuged at 12,000× *g* for 10 min at 2–8 °C. After discarding the supernatant, a volume of 1000 mL of 75% ethanol was added and the samples were again centrifuged at 7500× *g* for another 5 min at 4 °C. The ethanol was discarded, and after ensuring that all remaining solvent had evaporated, 30 mL of ddH_2_O was added to dissolve the RNA. The resulting RNA samples were incubated on ice for 15–20 min and then quantified using a NanoDrop 100 (Thermo Scientific, Waltham, MA, USA). The RNA OD value was detected by Scandrop100 using the A260/A280 ratio, and first-strand cDNA was synthesized using the TRUEscript 1st Strand cDNA Synthesis Kit (Aidlab Biotechnologies Co., Ltd., Beijing, China) according to the manufacturer’s instructions.

### 2.3. Construction of Illumina cDNA Library and Sequencing

Before subsequent experiments, the total RNA was first processed for mRNA enrichment and rRNA removal. In the first case, Oligo(dT) magnetic beads were used to enrich for mRNA with polyA tails. The rRNA was then removed by initial hybridization with a DNA probe and RNaseH was used to the selectively digest DNA/RNA hybridized chains, before eventually digesting the DNA probe with DNaseI. After purification, the required RNA was then obtained. An appropriate amount of interrupting reagent was added to cause the mRNA to fragment at high temperatures, and first-strand cDNA was synthesized using the fragmented mRNA as the template. The two-chain synthesis reaction system was then configured to synthesize two-chain cDNA, and the kit used to recover and purify the sticky-end cDNA 3′ ends, and repair them by joining them to base “A”. After fragment size selection, PCR amplification was carried out. The constructed libraries were inspected by the Agilent 2100 Bioanalyzer (Agilent Technologies, Inc., Palo Alto, CA, USA)and ABI StepOnePlus Real-Time PCR System (Applied Biosystems Inc., Mass, Waltham, MA, USA), prior to the transcriptomic sequencing of Yunwu Tribute tea in DW and CK groups on an Illumina HiSeq platform (Illumina, San Diego, CA, USA).

### 2.4. Functional Annotation and Classification

Using BLASTx (accessed on 15 January 2022), unigene sequences were aligned to the KEGG, GO, NR, NT, SwissProt, Pfam and KOG databases (E-value ≤ 10) and the proteins with the highest sequence similarity with the given unigenes were selected to obtain information on the functional annotation of the sequences. In addition, the sequences themselves were matched to the non-redundant nucleotide (NT) database using BLASTn in order to extract information on the species to which the sequences belonged. The unigenes and KOG databases were also compared to predict functions and make functional classifications.

### 2.5. Predictive Analysis of Encoding Protein Frames (CDS) and Transcription Factors

Transdecoder, the software recommended by Trinity, was used to identify candidate coding regions in unigene sequences. The longest open reading frame was first extracted using LongOrfs (accessed on 15 January 2022), and the fasta sequences were then compared with results from the SwissProt database using Diamond Blastp (accessed on 25 January 2022). Hmmscan was then used to filter Blast results to identify Pfam protein homologous sequences in order to predict coding regions using TransDecoder (accessed on 25 January 2022). 

TF, short for transcription factor, is a group of protein molecules that can bind to a specific sequence upstream of the 5′ end of a gene to ensure that the target gene is expressed at a specific intensity as well as at a specific time and space. Getorf was used to detect the ORF of unigenes, before using HMMSearch to compare the ORF to the transcription factor protein domain (data from TF). The ability of unigenes to bind TF was determined based on the characteristics of transcription factor families as described by PlantTFDB.

### 2.6. GO and KEGG Analysis of Differentially Expressed Genes

Reads indicating possible contamination as well as those of low quality and containing unknown bases (N) were filtered out to obtain called clean reads which were subsequently assembled to obtain unigenes. This was followed by the functional annotation of the unigenes using Bowtie2 [5,6] by comparing the clean reads to reference gene sequences, and RSEM was then used to calculate the expression levels of genes and transcripts. For multiple samples, the differentially expressed genes between them were detected according to their needs, and in-depth cluster analysis as well as functional enrichment analysis were performed for the differential expressed genes.

Based on GO and KEGG annotation results as well as curated classification data, the differential genes were classified according to functional and biological pathways, with the Phyper function in R also used for enrichment analysis. The *p*-values were calculated as shown below, and subsequently corrected for FDR (False Discovery Rate) to obtain the q-value. Generally, q-values ≤ 0.05 are regarded as an indication of significant enrichment.

$$P=1-\overset{m-1}{\underset{i=0}{\sum }}\frac{\left(\begin{array}{c} M\\ i \end{array}\right)\left(\begin{array}{c} N-M\\ n-i \end{array}\right)}{\left(\begin{array}{c} N\\ n \end{array}\right)}$$

### 2.7. Validation of RNA-Seq Data by Quantitative Real-Time PCR

Although RNA-Seq remains a powerful tool for screening differentially expressed genes, errors can still exist in the transcriptome data assembled from billions of short reads. Therefore, in order to verify the effectiveness of the unigene expression profiles obtained, five cold-tolerance related genes were selected for quantitative real-time polymerase chain reaction (qRT-PCR).

For the qRT-PCR, the primers (Table 1) were designed by Beacon Designer 7.9 (Premier Biosoft International, Diamond Heights Blvd, San Francisco, CA, USA) and synthesized by Chengdu Danfeng Technology Co., Ltd. (Chengdu, China). The amplification curve and melting peaks of each primer are shown in Supplementary Figures S1–S6. After adding all the components, the tubes were centrifuged at 6000 rpm for 1 min to keep all the components at the bottom and the reactions were then carried out as follows: 95 °C for 3 min; 95 °C for 10 s; 58 °C for 30 s + plate read; and 39 cycles, each at 95 °C for 10 s. 

### 2.8. Statistical Analysis

The relative expression of target genes in each sample was automatically calculated using the qPCRsoft 3.2s and Pfaffl methods simultaneously according to the following equation:

$$\mathrm{Ratio}=\frac{{\left(1+E_{\mathrm{taget}}\right)}^{{\Delta \mathrm{Ct}}_{\mathrm{target}\;\left(\mathrm{control}-\mathrm{expt}\right)}}\;}{{\left(1+E_{\mathrm{reference}}\right)}^{{\Delta \mathrm{Ct}}_{\mathrm{reference}\;\left(\mathrm{control}-\mathrm{expt}\right)}}\;}$$

The relative gene expression of each sample was calculated using 2-DDCt with actin as the internal reference.

A one-way analysis of variance (ANOVA), followed by a post hoc Fisher’s Least Significant Difference test, was used to examine significant differences between measurements. Differences between samples in each treatment (*p* < 0.05) were indicated by different letters, while values with same letter did not differ from each other.

## 3. Results

### 3.1. Analysis of RNA-Seq and Splicing Results

The Illumina HiSeq platform which was used to sequence two libraries (DW cryogenic group and CK control group) generated a total of 38.69 Gb of data. After assembly and the removal of redundancies, 185,973 unigenes were obtained, with their total length, average length, N50 and GC contents being 184,537,732 bp, 992 bp, 1894 bp and 40.36%, respectively (Table 2).

### 3.2. Functional Annotation and Classification of Unigenes

#### 3.2.1. Functional Annotation

The 185,973 unigenes were compared with seven functional databases (KEGG, GO, NR, NT, SwissProt, Pfam and KOG); overall, 113,627 (61.1%) unigenes matched at least one of the seven databases. More specifically, the number and percentage of unigenes which were functionally annotated for each database were as follows: 96,058 (NR: 51.65%), 92,509 (NT: 49.74%), 71,138 (SwissProt: 38.25%), 76,354 (KOG: 41.06%), 74,417 (KEGG: 40.01%), 53,390 (GO: 28.71%) and 68,067 (Pfam: 36.60%). In addition, of all the unigenes, 28,935 (15.56%) had matches within all seven databases (Table 3).

#### 3.2.2. KOG Functional Classification

**Figure 1:**
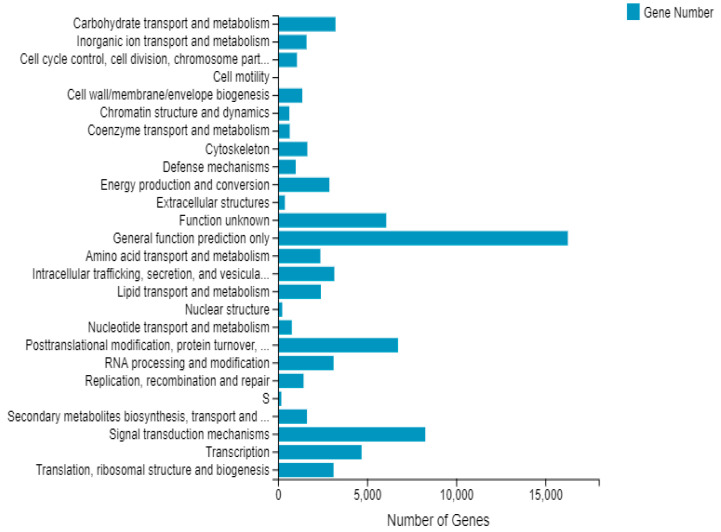
Functional classification of KOG.The *X*-axis represents the corresponding number of Unigene and the *Y*-axis represents the name of KOG functional classification.

After comparing unigenes with the KOG database, a total of 76,354 unigenes were annotated into 25 categories. The results, shown in Figure 1, indicated that most of them (16,266 unigenes corresponding to 21.3%) were predicted as having general functions. Similarly, 8265 unigenes (10.8%) were predicted as being involved in signal transduction while 6728 of them (8.8%) were considered to take part in posttranslational modification, protein turnover, and chaperone roles. 6074 of the unigenes (8.0%) were also identified as having unknown functions. In addition, 18 categories had more than 1000 annotated unigenes, while the number of unigenes involved in extracellular structures and nuclear structures were relatively small at 384 (0.5%) and 237 (0.3%), respectively (Figure 1).

#### 3.2.3. Species Annotated Classification

**Figure 2:**
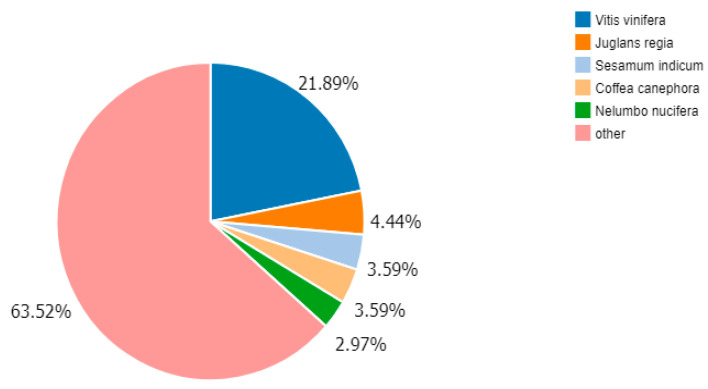
Characteristics of homology search of query sequences aligned by BLASTx to the Nr database. Species distribution of the first BLAST hits for each sequence with a cutoff E-value of 1.0E-5.

Based on the identification of annotated species, most of the unigenes (accounting for 63.52% of the total) were annotated to species classified as “other”. This was followed by *Vitis vinifera*, which corresponded to 21.89%, while *Juglans regia*, *Sesamum indicum*, *Coffea canephora* and *Nelumbo nucifera* accounted for 4.44%, 3.59%, 3.59%, and 2.97%, respectively (Figure 2).

#### 3.2.4. GO Function Classification

**Figure 3:**
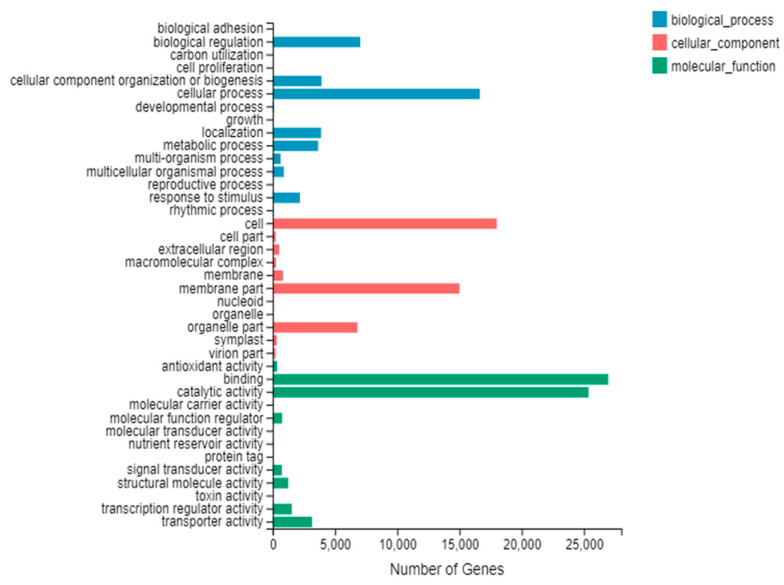
Histogram presentation of Gene Ontology classifications.

All the unigene results, previously compared with the NR database, were annotated according to the GO database, with three statistically significant groups identified, namely Biological Process, Cellular Component and Molecular Function (Figure 3). A total of 15 GO classes were identified as Biological Process, 11 as Cellular Component, and 13 as Molecular Function.The largest categories for biological processes consisted of cellular processes (16,636), biological regulation (7033) and cellular component organization or biogenesis (3913). With regard to the cellular component, the largest categories were cell (17,996), membrane (15,002) and organelle (6806). Finally, the largest groups identified in the case of molecular function were binding (26,936) and catalytic activity (25,355). Among these, five categories, namely cell process, cell, membrane, binding and catalytic activity were highly enriched, probably due to the vigorous metabolic activities in the leaves of Yunwu Tribute tea in response to low temperatures.

### 3.3. Prediction of Genes Encoding Transcription Factors

**Figure 4:**
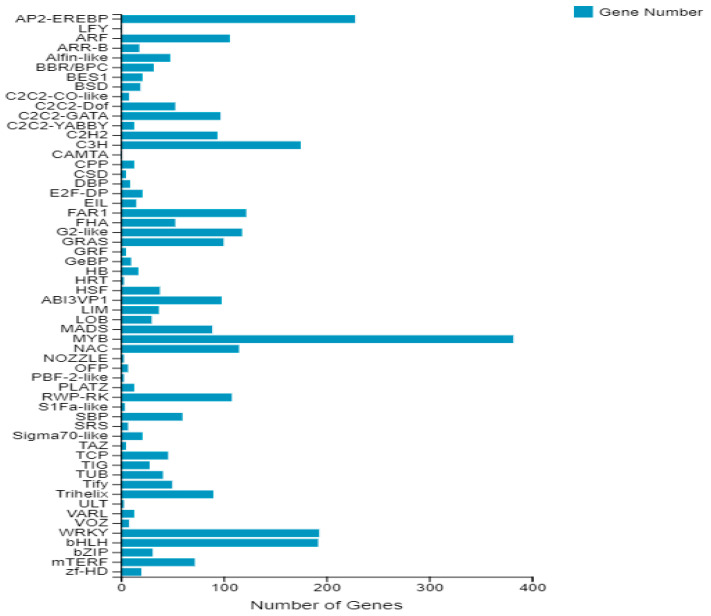
DEGs from every gene family involved transcription factors.

Genes with the ability to encode transcription factors (TF) were predicted, with the transcription factor families to which they belonged classified and statistically analyzed. The results, shown in Figure 4, indicated that a total of 3212 unigenes encoding for transcription factors were predicted which could be further divided into 58 families based on their putative DNA-binding and kinase domains. The MYB family had the largest number of unigenes (382), followed by the APETALA 2/ethylene responsive element-binding protein (AP2/ER EBP) at 228, WRKY at 193 and basic helix-loop-helix (bHLH) at 192.

### 3.4. Protein Coding Sequence Prediction

**Figure 5:**
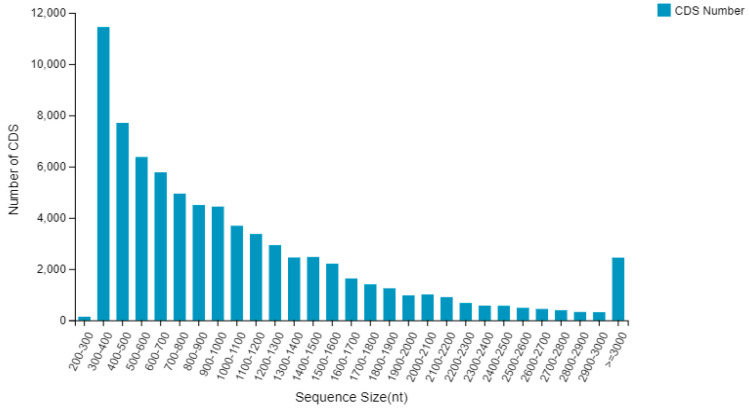
Length distribution of CDS.

Using BLASTx (accessed on 15 January 2022) to compare sequences with the Nr database, 75,830 CDS were detected by Transdecoder (accessed on 25 January 2022), with their total length being 80,863,683 bp, the maximum length being 15,333 bp, the minimum length being 297 bp, the N50 being 1377 bp, and the GC% being 44.38%. Among the coding sequences of these unigenes, a total of 56,540 (accounting for 74.56% of the unigenes) were greater than 500 bp, while 30,509 coding sequences, accounting for 40.23%, were greater than 1000 bp (Figure 5).

### 3.5. Analysis of Metabolic Pathways of the Differentially Expressed Genes

In order to better understand the biological functions of the unigenes, comparison between BLASTx results and the KEGG database (E-value ≤ 10) was performed to determine the biological pathways. To further clarify the biological functions of the differentially expressed genes, KEGG and pathway enrichment analyses of these genes were performed using the BINGO 3.0.2 (Biological Networks Gene Ontology tool) software.

**Figure 6:**
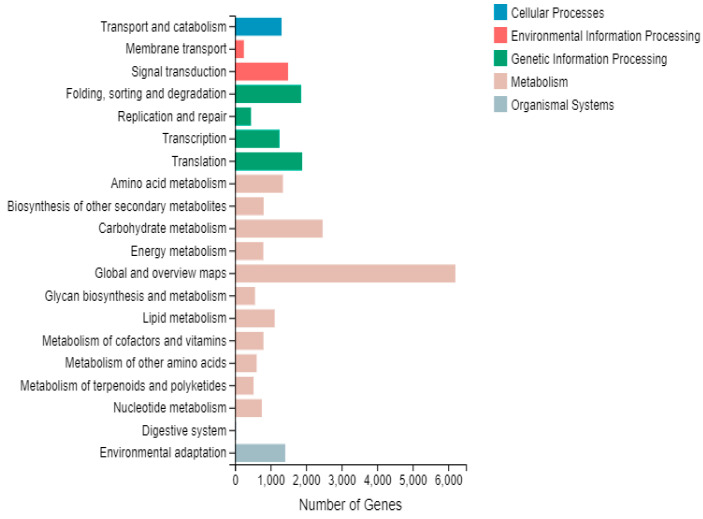
Category of KEGG annotation in differential gene.

A total of 14,516 differential genes were annotated according to five different groups of the KEGG metabolic pathway: cellular processes, environmental information processing, genetic information processing, metabolism, and organismal systems. The largest number of unigene annotations were found to be within the metabolic processes group, which involved a series of biochemical reactions related to metabolic processes such as carbohydrate metabolism, amino acid metabolism, lipid metabolism, secondary metabolite synthesis, energy synthesis, nucleotide metabolism and polysaccharide metabolism. Specifically, the unigenes involved in carbohydrate metabolism numbered 2466, those in translation 1889, while those in folding, sorting and degradation were 1858 (Figure 6).

**Figure 7:**
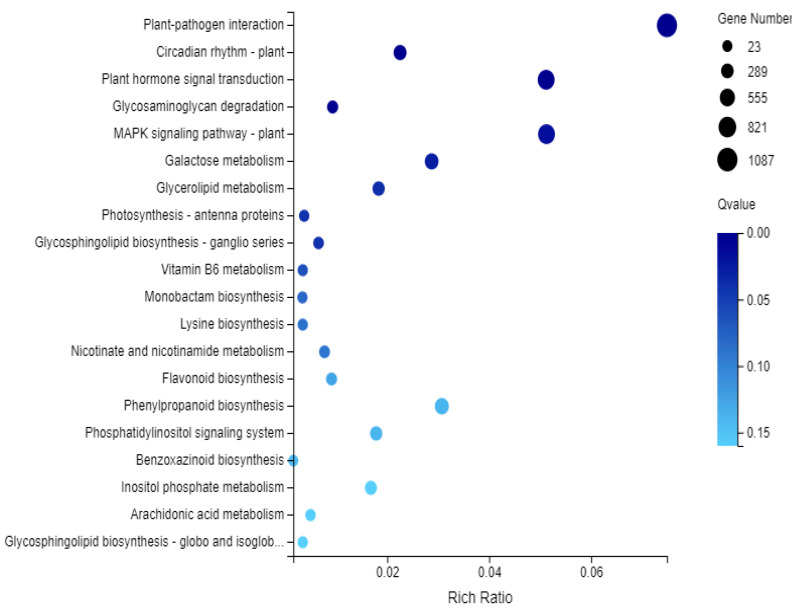
Enrichment bubble pattern of KEGG in differential gene.The horizontal axis of the bubble map of differential enriched KEGG Pathway shows the enrichment ratio, and the vertical axis shows the GO Term. The bubble size represents the number of differential enriched genes annotated to a certain KEGG Term, and the color represents the enriched Qvalue, and the darker the color represents the smaller the Qvalue.

KEGG enrichment analysis showed that 14,516 unigenes were involved in 135 KEGG pathways. Among these, the metabolic pathways (KO01100) contained most of the unigenes (5845). This was followed by the biosynthesis of secondary metabolites (KO01110) and plant-pathogen interactions (KO04626), to which 3078 and 1087 unigenes were respectively assigned (Figure 7).

#### 3.5.1. Peroxisome Metabolic Pathway

The concentration of reactive oxygen free radicals normally increases after a tea plant is placed under stress at low temperatures. Since a large amount of accumulated oxygen free radicals can damage both the cell membrane and the plant, the enzyme system in the plant body will act to reduce and eliminate those reactive oxygen species, thus playing an important role in cold tolerance. In this context, changes in the expression of the following genes were identified:

Three genes were down-regulated in FAR (KO number 13356) of the membrane proteins.

Eight genes were up-regulated and two were down-regulated in CAT (KO number 03781) of the antioxidant system. Similarly, eleven genes were up-regulated and twenty were down-regulated in SOD (KO number 04564). One gene was also up-regulated in PRDX5 (KO number 11187).

In the unsaturated fatty acid β-oxidation system, one gene was up-regulated and another one was down-regulated in PDCR (KO number 13237) while sixteen genes were up-regulated and twenty-seven were down-regulated in ACSL (KO number 01897).

In the amino acid metabolism PTS1 type system, three genes were up-regulated while one was down-regulated in AGT (KO number 00830). For IDH, nine genes were up-regulated and fourteen were down-regulated (KO number 11517), while six genes were up-regulated and seventeen were down-regulated in the case of HAO (KO number 11517).

In the retinol metabolism system, two genes were up-regulated in DHRS4 (KO number 11147).

In the ROS metabolism system, three genes were up-regulated and four were down-regulated in PXMP2 (KO number 13347). Similarly, fourteen genes were up-regulated and twelve were down-regulated in MPV17 (KO number 13348).

**Figure 8:**
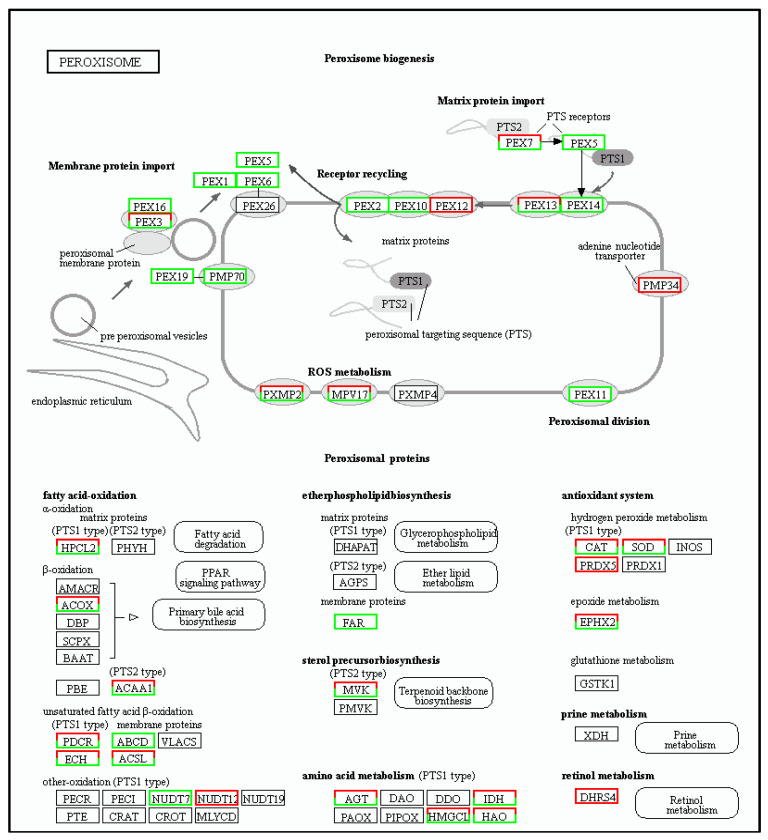
The cold tolerance genes of tea plants involved in peroxisome pathways. Red boxes representing the upregulation of genes and green ones representing their downregulation.

For the peroxisome metabolic pathway, 332 differentially expressed genes were identified, of which 123 genes were up-regulated and 209 genes were down-regulated. These results are summarized in Figure 8.

#### 3.5.2. Proline Metabolism Pathway

**Figure 9:**
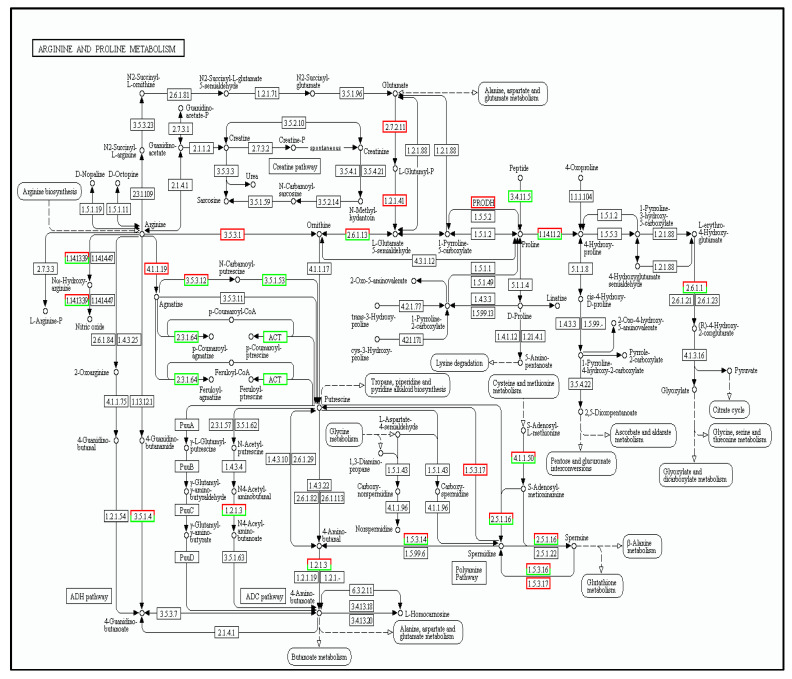
The cold tolerance genes of tea plants involved in proline metabolism pathways. Red boxes representing the upregulation of genes and green ones representing their downregulation.

In this metabolic pathway, glutamate goes through 2.7.2.11 to Pyrrolin-5- carboxylic acid synthetase (P5CS), and six genes were up-regulated in this case (KO number 12657). As shown in Figure 9, there were 197 differentially expressed genes (DEGs) in the proline metabolism pathway, of which 111 genes were up-regulated and 86 were down-regulated.

#### 3.5.3. Tyrosine Metabolism Pathway

**Figure 10:**
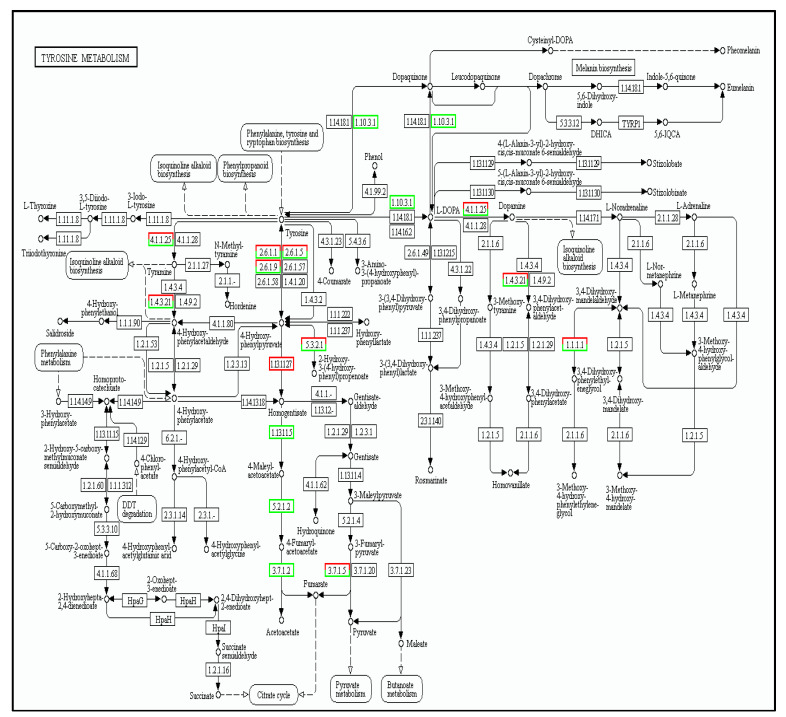
The cold tolerance genes of tea plants involved in Tyrosine metabolism pathways. Red boxes representing the upregulation of genes and green ones representing their downregulation.

In this metabolic pathway, tyrosine goes through 1.10.3.1 to synthesize dopaquinone; for this process, three genes were down-regulated (KO number 00422). As shown in Figure 10, 134 differentially expressed genes (DEGs) were identified for the tyrosine metabolism pathway; from these, 39 genes were up-regulated and 95 were down-regulated.

#### 3.5.4. Plant Hormone Signal Transduction Pathway

In plants, auxins and cytokinines promote plant growth and development. In the auxin metabolic pathway, the following changes in gene expression were noted:

One gene was up-regulated and another one was down-regulated in AUX1 (KO number 13946). One gene was up-regulated and ten were down-regulated in TIR1 (KO number 14485).

Eight genes were up-regulated and twelves genes were down-regulated in AUXLAA (KO number 14484). Twenty-three genes were up-regulated and twenty-one were down-regulated in ARF (KO number 14486).

One gene was up-regulated and seven were down-regulated in GH3 (KO number 14487).

Seven genes were up-regulated and thirteen genes were down-regulated in SAUR (KO number 14488).

Similarly, in the cytokinine metabolic pathway, changes in gene expressions were as follows:

One gene was up-regulated and twelve genes were down-regulated in CRE1 (KO number 14489). One gene was up-regulated and two were down-regulated in AHP (KO number 14490). Twenty-five genes were up-regulated and thirty-eight genes were down-regulated in B-ARR (KO number 14491).

Two genes were up-regulated and twelve were down-regulated in A-ARR (KO number 14492).

In the gibberellin metabolic pathway, ten genes were up-regulated and seven were down-regulated in GID1 (KO number 14493). For GID2 (KO number 14495), one gene was up-regulated and two were down-regulated, while twenty-six genes were up-regulated and eleven were down-regulated in the case of DELLA (KO number 14494).

The abscisic acid content in plants is also important for improving cold tolerance in plants. In this case, for the abscisic acid metabolic pathway:

Two genes were up-regulated and four genes were down-regulated in PYR/PYL (KO number 14496). Fourteen genes were up-regulated and five were down-regulated in PP2C (KO number 14497). Ten genes were up-regulated and another ten were down-regulated in SnRK2 (KO number 14498).Eight genes were up-regulated and ten were down-regulated in ABF (KO number 14432).

Under stress conditions, it was expected that the ethylene content of plants would also change. As such, it was observed that for the ethylene metabolic pathway:

Three genes were up-regulated and ten were down-regulated in ETR (KO number 14509). Four genes were up-regulated and fifteen were down-regulated in CTR1 (KO number 14510). Seven genes were up-regulated and four were down-regulated in EBF1/2 (KO number 14515). Eight genes were up-regulated and two were down-regulated in EIN3 (KO number 14514).Six genes were up-regulated and one gene was down-regulated in ERF1/2 (KO number 14516/14517).

In the case of the Brassinosteroid metabolic pathway:

Two genes were up-regulated and fifteen genes were down-regulated in BAK1 (KO number 13416). Twenty-three genes were up-regulated and another twenty-three were down-regulated in BRI1 (KO number 13415. One gene was down-regulated in BKI1 (KO number 14499). Thirteen genes were up-regulated and fifteen were down-regulated in BSK (KO number 14500). Six genes were up-regulated and three were down-regulated in BZR1/2 (KO number 14503). Six genes were up-regulated and three genes were down-regulated in TCH4 (KO number 14504). One gene was up-regulated and four genes were down-regulated in CYCD3 (KO number 14505).

Fat and lipid substances are also positively correlated with cold tolerance as they can prevent water exosmosis and reduce cell metabolism. In the jasmonic acid metabolic pathway:

Seven genes were down-regulated in JAR1 (KO number 114506). Four genes were down-regulated in COI1 (KO number 13463). Six genes were up-regulated and three were down-regulated in JAZ (KO number 13464). Twenty genes were up-regulated and forty-seven genes were down-regulated in MYC2 (KO number 13422).

Finally, in the salicylic acid metabolic pathway:

Eighteen genes were up-regulated and fourteen were down-regulated in TGA (KO number 14431). Eight genes were up-regulated and five genes were down-regulated in NPR1 (KO number 14508). One gene was up-regulated and four genes were down-regulated in PR-1 (KO number 13449).

**Figure 11:**
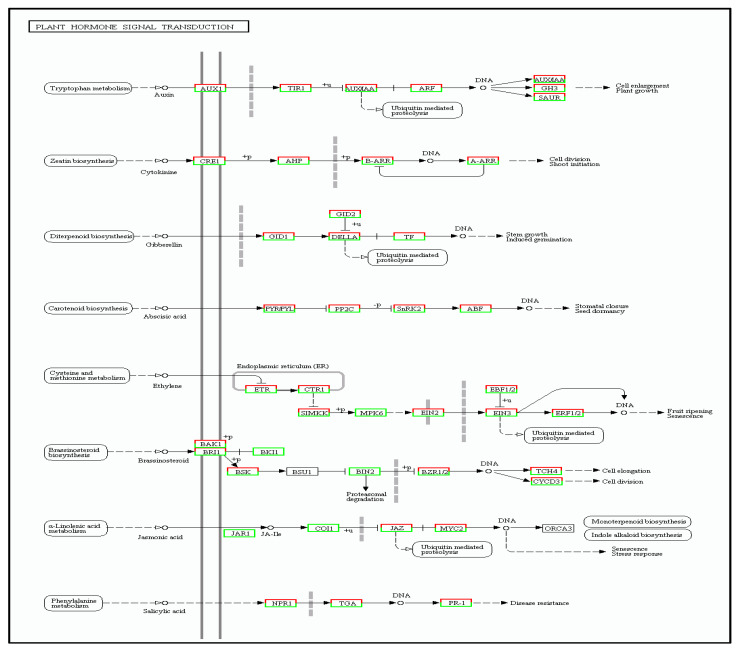
The cold tolerance genes of tea plants involved in metabolism of plant hormone signal transduction pathways.Red boxes representing the upregulation of genes and green ones representing their downregulation.

The results for the above differentially expressed genes of the plant hormone transduction pathway are summarized in Figure 11. Overall, there were 743 genes, of which 316 were up-regulated and 427 were down-regulated.

### 3.6. DEG Validation by qRT-PCR Analysis

**Figure 12:**
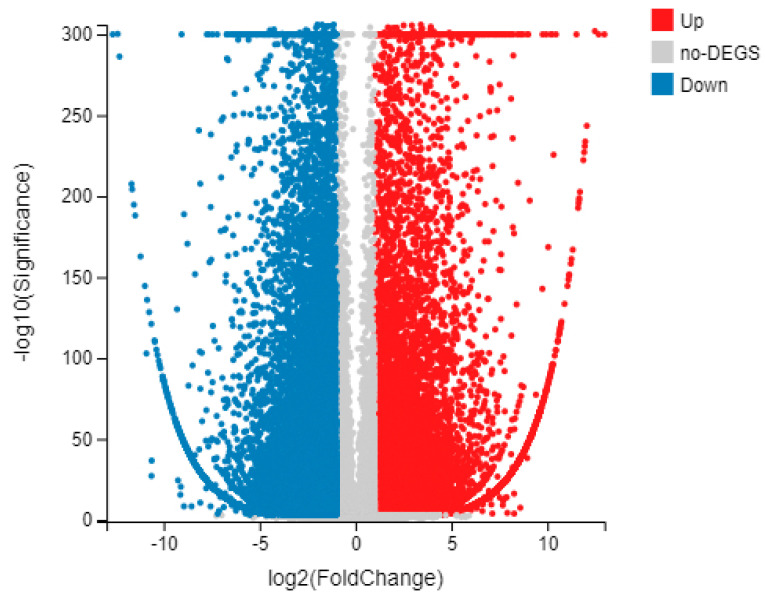
Volcanic map of inter-group differences. Each dot represents a gene, and the color is used to distinguish whether the gene is differential expressed. In the figure, the red dot represents the up-regulated differential expressed gene, the blue dot represents the down-regulated differential expressed gene, and the gray dot represents the non-differential expressed gene.

All of the 16,020 differentially expressed genes (DEGs) (corrected *p*-value < 0.01, |log2(fold-change)| > 3), including the 9606 up-regulated and 6414 down-regulated genes, were obtained by qRT-PCR. A volcanic map of inter-group differential genes can be used to reflect the overall differences in gene expression, with the horizontal and vertical axes representing log2 (fold-change), and −log10 (the smaller the *p* value, the more significant), respectively (Figure 12).

**Figure 13:**
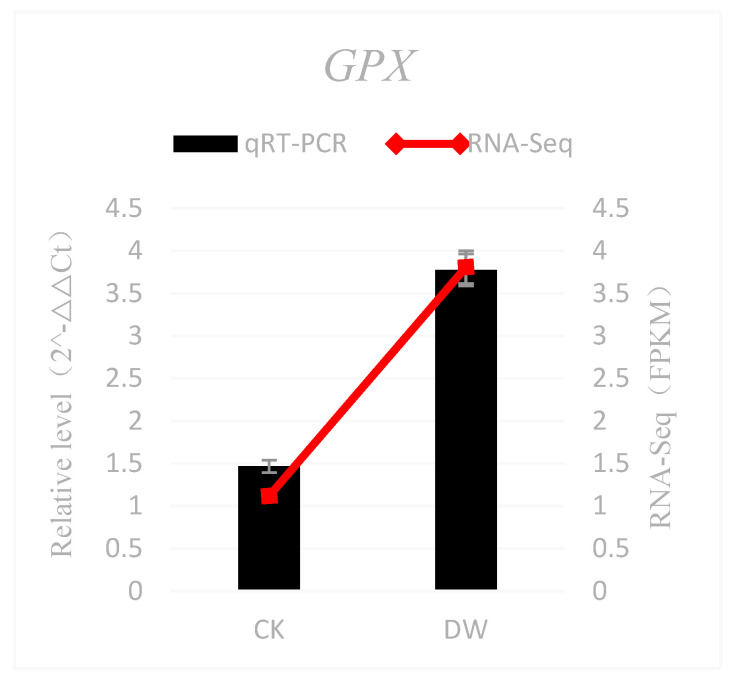

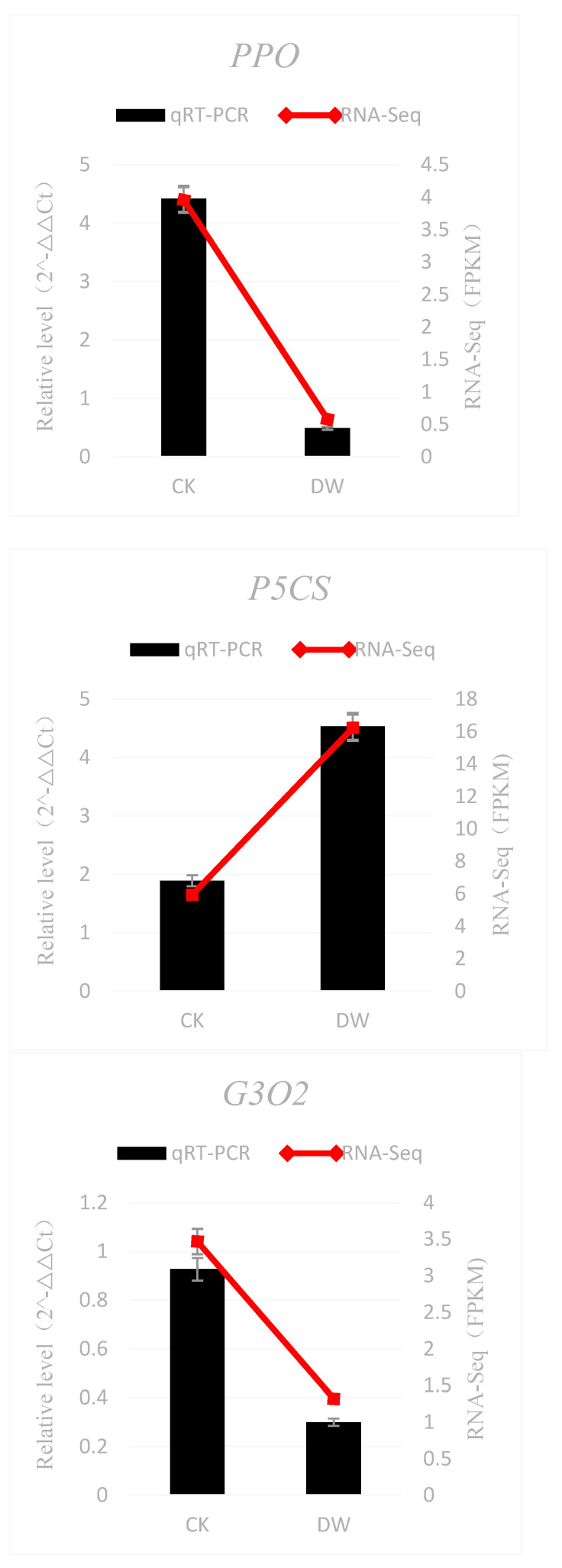

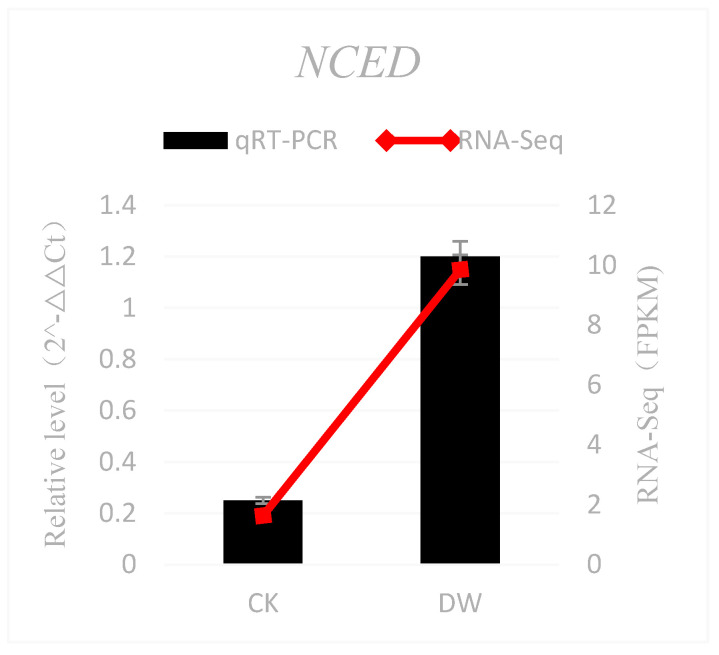
Validation of RNA-Seq results using qRT-PCR. CK is the control group and DW is the cold-treated group: (CK: 25 °C; DW: 1 °C).

For the Yunwu Tribute Tea, under cold stress, the expression levels of *GPX*, *P5CS*, and *NCED* were significantly up-regulated while those of *PPO* and *G3O2* were significantly down-regulated (Figure 13). In fact, the results showed that the expression patterns for these genes were consistent with those obtained by qRT-PCR and RNA-Seq.

## 4. Discussion

### 4.1. Molecular Mechanism of Response to Cold Stress

Under cold stress, the plant can respond in multiple ways, which include the adaptation of leaf and tissue structures, the alteration of cell membrane compositions, the rearrangement of cells, an increase in osmosis-maintaining substances (e.g., soluble sugar, proline, betaine), and an increase in the synthesis of antioxidants as a result of enhanced catalytical activity of certain enzymes (e.g., superoxide dismutase, peroxidase, ascorbate reductase). These strategies can help the plant to regain lost components and to balance energy generation, thus enabling it to adapt and survive in cold environments [7]. At the physiological level, cold stress can induce the perception of cold, signaling and transcriptional regulation [8], which are mediated by specific molecular mechanisms. For this purpose, gene expression is adjusted via extensive signaling and other regulating mechanisms in order to fine-tune the metabolism and growth of the plant to allow adaptation to low temperatures [9].

#### 4.1.1. Perception of Low-Temperature Signal

The cellular membrane system consists of both the cell membrane and the endomembrane system. The cell membrane, representing the boundary where a cell interacts with the internal environment, is partially permeable to substances, and it maintains the equilibrium of a cell. On the other hand, the endomembrane system is the site where energy production as well as the synthesis, degradation and transfer of substances occur. As far as tolerance to low temperature is concerned, the stability of the cell membrane system is a key factor, as low-temperature stress can reduce the fluidity of the cellular membrane while increasing its permeability, causing soluble contents in the cells to diffuse out and resulting in metabolic disorders. At low temperatures, the cell membrane first senses the change in temperature, which then triggers appropriate physiological, biochemical and physical changes in the cell. For instance, within a certain low temperature range, fatty acid desaturases located on the cell membrane were found to be activated, causing large quantities of unsaturated fatty acids to be accumulated. This causes the fluidity of the membrane to be reduced, as the lipid membrane is changed from a disordered and liquid crystalline state to an ordered, gelatinous and partially solid one so that the stability of the cellular structure is maintained [10,11]. In fact, such is the contribution of the fatty acids to cold tolerance that it is considered that tolerance can be significantly improved by increasing the content of unsaturated fatty acids or the degree of fat unsaturation in plants [12,13,14]. In *Medicago satia* and *Brassica napus*, the rigidification of cell membrane using drugs induced the expression of COR (cold-responsive) genes, which were shown to be related to cold-acclimatization properties [15]. Furthermore, the selectiveness of the cell membrane was found to decline with a steady drop in temperature, causing solvents (e.g., K^+^, sugars and amino acids) to diffuse out. At the same time, ice cores were formed in intracellular spaces, and the plasma gelatinized in a non-reversible way. This caused the cells to freeze, thereby damaging the cell membrane, disturbing the cell metabolism and affecting the overall growth of the plants [16].

#### 4.1.2. Transduction of Low-Temperature Signal

After sensing a low temperature through receptors on the cell membrane, proteins (mainly G-proteins) re phosphorylated by phospholipases, thus activating the Ca^2+^ channel on the cell membrane and increasing the Ca^2+^ concentration in cells. In this way, the specific Ca^+^ signals triggered by the low temperature are transferred from the external environment to that of the internal cell membrane [9]. Currently, Ca^2+^ is regarded as a second messenger for the transfer of signals related to low temperature in plants as, based on its intracellular concentration, certain Ca^2 +^ -dependent protein kinases (CDPK) are phosphorylated or dephosphorylated, hence inducing the expression of genes related to low temperature. In this context, it has been reported that, when *Arabidopsis thaliana* L. or *Medicago satia* were exposed to low temperatures, Ca^2+^ stored in the surrounding environment flowed into the cells, causing its intracellular concentration to rise rapidly. This temporary surge of Ca^2+^ was indispensable for inducing the expression of genes related to cold acclimatization and cold tolerance [17]. Furthermore, in *Arabidopsis thaliana* L., the full expression of some low-temperature-regulated genes such as CRT/DRE (encoding cor 6.6) were also dependent on the increase of Ca^2+^ levels [18]. Similarly, in the case of seedlings of winter wheat and *Trititrigia*, training the plants for cold acclimatization promoted high activity of Ca^2+^ -ATPase on the cell membrane, so that Ca^2+^ equilibrium of the cells could be maintained [19]. Additionally, CDPK was also found to play a role in the signaling of the cold-stress-responsive mechanism while Saijo et al., [20] found that, under induced cold stress, OsCDPK were overexpressed in transgenic rice, which also displayed better salt- and drought- resistance.

However, in addition to Ca^2 +^, studies have suggested that the plant hormone abscisic acid (ABA) is also associated with low-temperature signaling [21]. Under normal growth conditions, by applying external ABA and therefore reducing the expression of cold-responsive genes, protein synthesis was facilitated, and cold tolerance was improved in plants [22,23]. Studies involving ABA-insensitive mutants (ABI) and ABA-synthesis-deficient mutants (ABA-deficient) of *Arabidopsis thaliana* L. demonstrated that cold acclimatization and cold tolerance decreased in both mutants. However, when supplemented with external ABA, cold-acclimatization properties were recovered in the ABA mutants [24,25,26]. Moreover, increasing evidence are suggesting that protein kinases and protein phosphorylases (PP) are also involved in the signaling of low-temperature acclimatization [27].

#### 4.1.3. Low-Temperature Transcriptional Regulation

In response to hormonal stimuli and external environmental stresses, transcriptional regulation controls the intensity of gene expression or regulates gene expression spatiotemporally through the specific binding of the transcriptional factor proteins to target genes. In this study, the low temperature resulted in drastic changes in the plant transcriptome. It is estimated that cold-regulated genes account for 4–20% of the genome in *Arabidopsis thaliana* L. [28,29]. When faced with low-temperature stress, the plant senses the cold signal and activates the pathway for downstream signaling. The transductors in the cell wall, the cell membrane and plasma amplify the cold signal in cascades and transfers it into the nucleus for inducing the expression of cold-regulated genes, which will enable the plants to acclimatize to the cold in a short period of time [30]. Owing to the variety of abiotic stress receptors and signaling pathways in plants, cold stress could, in fact, activate a number of different signaling paths, causing various expression results. Meanwhile, different receptors and paths for signal transduction are also present downstream of Ca^+^ and ABA [30]. Collectively, the transduction of stress signals in plants is a complex network, involving interactions between the signals attributed to the multi-gene regulation after environmental stimulation. Several cis-acting elements often locate the promoter of a single cold-regulated gene so that a gene may be regulated by multiple signaling systems rather than being dependent on only a single or one type of signaling molecule. For instance, *cis*-acting elements of the ABA response element (ABRE) and dehydration-responsive element (DRE) were present in the promoters of those genes whose expression were induced by drought, high salinity, and low temperature, namely RD29A, COR78 and LTI 78, suggesting that the expression of these genes was simultaneously regulated by both ABA-dependent and non-ABA-dependent signaling pathways [31].

### 4.2. Application of RNA-Seq in Plants

The second generation of RNA-Seq has been widely applied in many aspects of plant research, primarily for studying the differences between developmental stages, tissues and organs, mutants, and plant characteristics under different environments. Gene expression analysis, DEG identification, mining of functional genes, and phylogenetic analysis of these different conditions have been achieved with the help of transcriptome sequencing [32]. Currently, RNA-seq has revealed the cold-stress-responsive mechanism of dozens of economically important crops, including *Arabidopsis thaliana* L., *Oryza sativa* L., *Brassica napus* L., *Camellia sinensis*, *Triticum aestivum* L., *Nicotiana tabacum,* and *Sorghum bicolor* L. This approach not only provides insight into the molecular mechanism of cold tolerance and the breeding of cold-tolerant strains, but also provides a technological reference and basic information for the stress-tolerance or avoidance mechanisms in other species [33]. As RNA-Seq technologies continue to be upgraded, and analytical methods improve, it is expected that RNA-Seq will be more extensively and deeply applied in plant biology studies.

Transcriptome analysis has promoted studies on the cold tolerance of tea plants, with the information available on a number of cold-related genes. Ref. [34] analyzed the transcriptomes of tea at different stages of cold acclimatization in the wild, and obtained 1770 DEGs, including those corresponding to cold signal transduction and responsive factors, cell membrane-stabilizing genes and osmosis-responsive genes. Further analyses also demonstrated the crucial role of carbohydrate metabolism and Ca^2+^ signal transduction in the cold tolerance of tea, with these results providing an important reference for further studies on the cold tolerance of tea plants. In addition, based on transcriptome data, more genes related to cold tolerance in tea have been cloned, e.g., the key genes in the betaine synthesis pathway [35] and genes encoding glutathione reductases (CsGRs) [36] analyzed the mechanisms regulating the biosynthesis of secondary metabolites, particularly catechin, caffeine and theanine, for different tissues of tea plants via RNA-Seq. Similarly, ref. [37] demonstrated the molecular mechanism responsible for differences in catechin content of the leaves of different varieties of tea trees via RNA-seq. Ref. [38] also applied this method for elucidating the molecular mechanism of the influence of flavonoid 3’-hydroxylase and flavonoid 3’,S’-hydroxylase genes on the ratio of dehydroxylated catechins to trihydroxylated catechins under different shading treatments. Likewise, ref. [39] compared differences between the transcriptomes of the Fudingdabai and Xiaoxueya tea-breed leaves, to explore the possible molecular genetic mechanism of inducing albino shoots in the Xiaoxueya breed.

With the development and application of modern gene technology, researchers have studied the regulation of gene expression for cold tolerance of tea trees. This has provided theoretical support for the clarification of cold-tolerance mechanisms of tea trees and the breeding of cold-tolerant varieties. With further research, transcriptomic analysis will be more widely used in the study of tea plants, and its combined analysis with other omics such as proteomics and metabolomics will help to better understand and clarify the physiological and biochemical mechanisms of tea plants.

## 5. Conclusions

In this study of Yunwu Tribute Tea under low temperature, a total of 185,973 unigenes were acquired using the Illumina HiSeq technology. From these, 16,020 DEGs (9606 up-regulated and 6414 down-regulated) were identified (corrected *p*-value < 0.01, |log2(fold-change)| > 3) in Yunwu Tribute Tea. It was found that substantial cold-responsive genes were related to the antioxidant enzyme system, plant hormone signal transduction, proline and tyrosine metabolism pathways, and transcription factors. Although the cold-resistance mechanism in Yunwu Tribute Tea could not be fully explained by our preliminary study, these potential cold-resistant genes offer the possibility of improving low-temperature tolerance in Yunwu Tribute Tea on a large scale. It is expected that this study will help to further understand the cold-responsive molecular mechanism of Yunwu Tribute Tea.

## Figures and Tables

**Table 1:** Forward and Reverse Primers Used for qRT-PCR Reactions.

*Gene Name*	Nr Description	Primers
*GPX*	Glutathione oxidase	F-CTGAGTGGAGAAGTAGTG
		R-GTGATGGAATTAAGTGGAAT
*PPO*	Polyphenol oxidase	F-CACAACCAACCTTCCCAACAAA
		R-TGCTTCTTGATTTCTTCGGTCTCT
*P5CS*	Delta-1-pyrroline-5-carboxylate synthase	F-ATTGTTGATGATGTGTATGC
		R-GGTCTTCTGTGATAATGCTA
*NCED*	9-cis-epoxy carotenoid dioxygenase	F-GTAAGTCTCTGCTGTAAC
		R-CTGTCTCAATTCACTCTC
*G3O2*	Gibberellin 3β dioxygenase	F-CCTATGTTGACCACGAGAG
		R-CGACCCTACTCACCATTC
*ACTIN*	*ACTIN*	F-GTATCGCAGACCGTATGAG
		R-TCCTCCAATCCAGACACT

**Table 2:** Overview of Sequencing and Splicing Data.

Sequences	CK1	CK2	CK3	DW1	DW2	DW3	Total
Total Raw Reads (M)	45.87	45.87	44.23	44.23	45.87	45.87	90.65
Total Clean Reads (M)	43.47	43.44	42.2	42.07	43.32	43.38	85.96
Total Clean Bases (Gb)	6.52	6.52	6.33	6.31	6.5	6.51	12.9
Clean Reads Q20 (%)	98.94	98.92	99.04	98.91	98.85	98.89	98.93
Total Mapping (%)	81.19	79.41	82.77	80.3	75.56	79.01	79.7
Total Number of Unigenes (bp)	78,914	82,358	74,522	81,326	103,803	91,064	185,973
Total Length of Unigenes (bp)	71,564,717	76,190,685	76,249,552	78,873,183	82,632,309	82,235,642	184,537,732
Mean Length of Unigenes(bp)	906	925	1023	969	796	903	992
N50 of Unigenes (bp)	1555	1586	1678	1644	1418	1591	1894
Unigenes GC (%)	41.34	41.14	41.48	41.5	40.88	41.09	40.36

**Table 3:** General Annotation List of Gene Function.

Values	NR	NT	Swissprot	KEGG	KOG	Pfam	GO	Intersection	Overall
Number	96,058	92,509	71,138	74,417	76,354	68,067	53,390	28,935	113,627
Percentage	51.65%	49.74%	38.25%	40.01%	41.06%	36.60%	28.71%	15.56%	61.10%

## Data Availability

Address for uploading transcriptome data: https://www.ncbi.nlm.nih.gov/sra/PRJNA784712 (accessed on 30 November 2021).

## Supplementary Materials

The following supporting information can be downloaded at: https://www.mdpi.com/article/10.3390/cimb45010047/s1, Figures S1–S6: The image of target gene.
