# Supplementary material for: Transcriptomic Analysis of Yunwu Tribute Tea Leaves under Cold Stress
Source: Curr Issues Mol Biol. 2023 Jan 13;45(1):699–720. doi: 10.3390/cimb45010047 (PMC9857825; doi:10.3390/cimb45010047)
Supplement: Supplementary file 1 [file cimb-45-00047-s001.zip › cimb-2083673-supplementary.pdf]

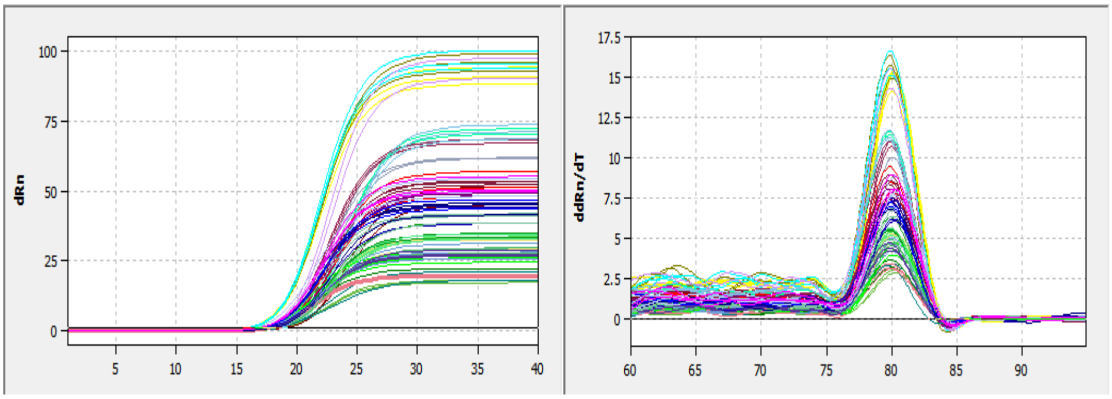

Figure S1 The image of internal reference ACTIN gene. The left is the amplification curve,abscissa is the number of cycles.The right is the melting peaks, abscissa is the dissolution temperature,the product TM value is 80℃.

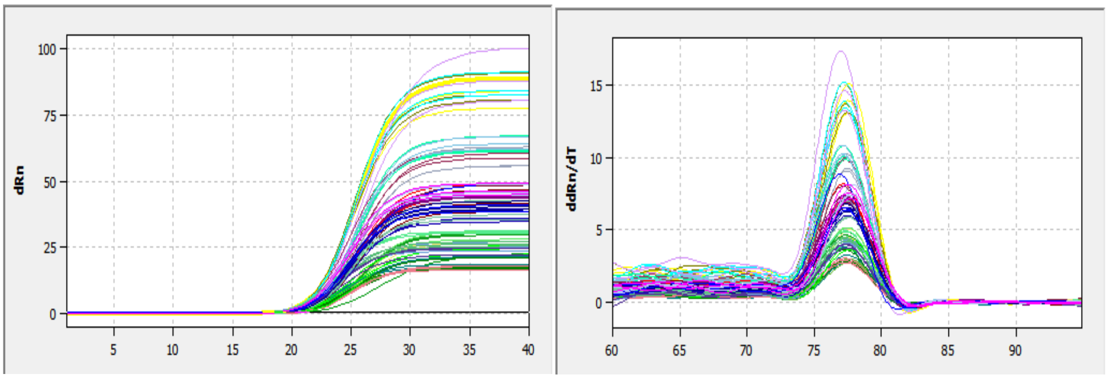

Figure S2 The image of target gene GPX. The left is the amplification curve,abscissa is the number of cycles.The right is the melting peaks, abscissa is the dissolution temperature,the product TM value is 77℃.

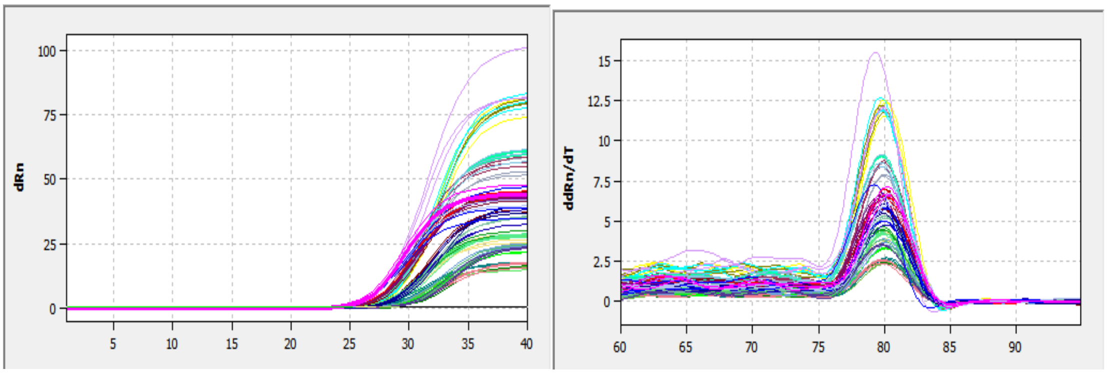

Figure S3 The image of target gene PPO. The left is the amplification curve,abscissa is the number of cycles.The right is the melting peaks, abscissa is the dissolution temperature,the product TM value is 80℃.

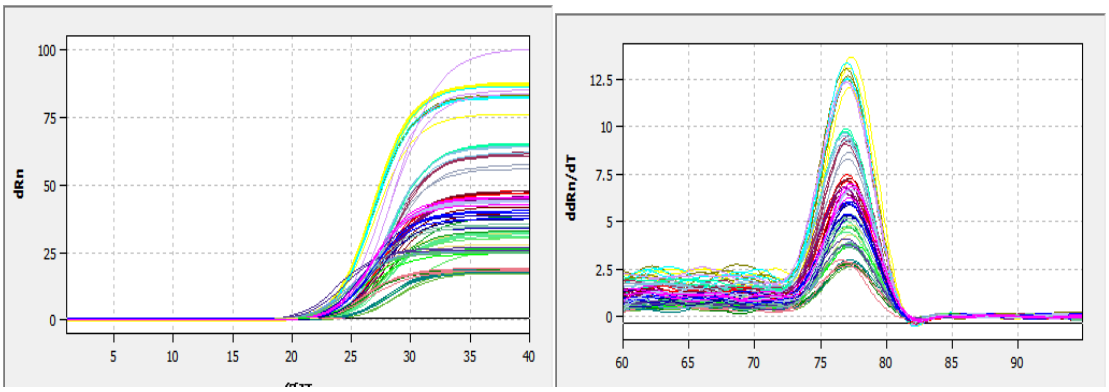

Figure S4 The image of target gene P5CS. The left is the amplification curve,abscissa is the number of cycles.The right is the melting peaks, abscissa is the dissolution temperature,the product TM value is 76.5℃.

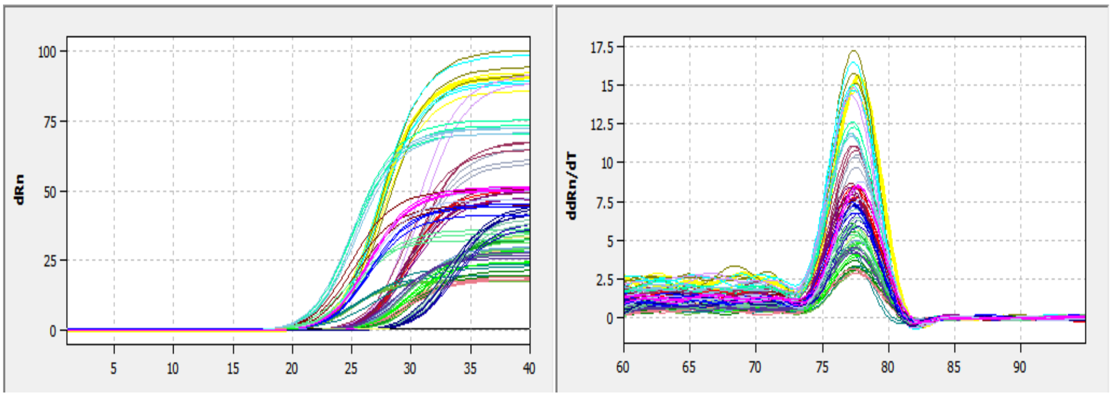

Figure S5 The image of target gene NCED. The left is the amplification curve,abscissa is the number of cycles.The right is the melting peaks, abscissa is the dissolution temperature,the product TM value is 77℃.

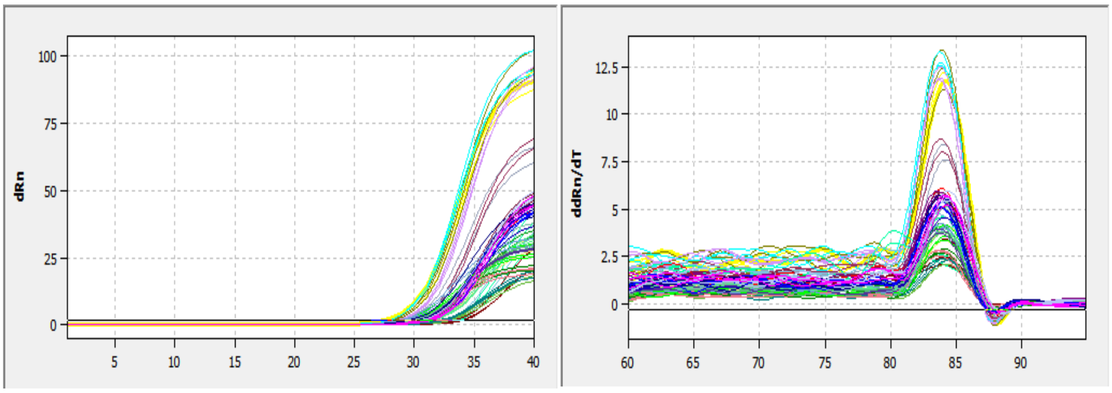

Figure S6 The image of target gene G3O2. The left is the amplification curve,abscissa is the number of cycles.The right is the melting peaks, abscissa is the dissolution temperature,the product TM value is 84℃.
